# Photonic quadrupole topological insulator using orbital-induced synthetic flux

**DOI:** 10.1038/s41467-022-33894-6

**Published:** 2022-11-03

**Authors:** Julian Schulz, Jiho Noh, Wladimir A. Benalcazar, Gaurav Bahl, Georg von Freymann

**Affiliations:** 1 Physics Department and Research Center OPTIMAS, TU Kaiserslautern, 67663 Kaiserslautern, Germany; 2 Department of Mechanical Science and Engineering, University of Illinois at Urbana-Champaign, Urbana, IL 61801 USA; 3 Department of Physics, Princeton University, Princeton, NJ 08542 USA; 4 Department of Physics, Emory University, Atlanta, GA 30322 USA; 5 Fraunhofer Institute for Industrial Mathematics ITWM, 67663 Kaiserslautern, Germany

**Keywords:** Optical materials and structures, Condensed-matter physics

## Abstract

The rich physical properties of multiatomic crystals are determined, to a significant extent, by the underlying geometry and connectivity of atomic orbitals. The mixing of orbitals with distinct parity representations, such as *s* and *p* orbitals, has been shown to be useful for generating systems that require alternating phase patterns, as with the sign of couplings within a lattice. Here we show that by breaking the symmetries of such mixed-orbital lattices, it is possible to generate synthetic magnetic flux threading the lattice. We use this insight to experimentally demonstrate quadrupole topological insulators in two-dimensional photonic lattices, leveraging both *s* and *p* orbital-type modes. We confirm the nontrivial quadrupole topology by observing the presence of protected zero-dimensional states, which are spatially confined to the corners, and by confirming that these states sit at mid-gap. Our approach is also applicable to a broader range of time-reversal-invariant synthetic materials that do not allow for tailored connectivity, and in which synthetic fluxes are essential.

## Abstract (web summary)

Here the authors experimentally demonstrate a photonic quadrupole topological insulator in a photonic lattice, in which required synthetic *π* flux is induced by different symmetry representations of the orbitals in a mixed-orbital lattice.

## Introduction

When studying materials and their physical properties, much emphasis is put on how atoms are combined to form molecules and crystalline structures through different orbital connections. In particular, the richness of macroscopic properties in multiatomic molecules and crystalline structures is closely related to the way in which orbitals connect. For example, a water molecule has an angled shape due to the hybridization of *s* and *p* orbitals, which in turn, causes the sixfold rotational symmetry of snowflakes. Similarly, the electronic properties of monolayer 2D transition metal dichalcogenides can be deliberately tuned over a wide range, in part, due to the interplay between *d* orbitals on metal atoms and *p*_*z*_ orbitals on chalcogen atoms^1–3^. In this context, the theory of "topological quantum chemistry”^4^ has greatly advanced the understanding of the intimate relationship between electronic orbitals and topological phases in crystalline structures and has led to the realization that topologically nontrivial materials are much more common than previously thought.

The ability of synthetic systems to replicate, and in many cases extend, the properties of chemical compounds and crystalline structures has recently become of great interest. As in real materials, the orbital degree of freedom can also be incorporated into synthetic systems by using analogous wavefunctions with distinct nodal structures. Such synthetic multi-orbital systems have been demonstrated in polariton lattices^5,6^, photonic lattices^7–10^, and ultracold atoms in optical lattices^11–16^, with the possibility of negative^8,16^ and even complex-valued^10,17^ coupling coefficients. These features have made synthetic platforms well-suited to explore novel physics that typically are difficult to study in solid-state systems.

Due to their inherent robustness and potential for disorder-resilient technologies, topological phases in synthetic periodic platforms are a very active area of study. Notably, the initial demonstrations of higher-order topological phases^18^ were produced using synthetic materials^19–27^. A quadrupole topological insulator (QTI) is the first member of the multipole higher-order topological insulators, but is not straightforward to implement as it requires a *π* flux of synthetic magnetic field threading each plaquette in the lattice^18,28^. In previous experimental realizations of QTIs^20–24^, the *π* flux was achieved by tailored connectivities within the system^20–23^ or couplings with arbitrary phases by exploiting additional coupling links^24^. However, these approaches are not always practical, especially in nanoscale geometries, and a good solution is needed to enable synthetic fluxes in a broader range of experimental platforms.

**Fig. 1:**
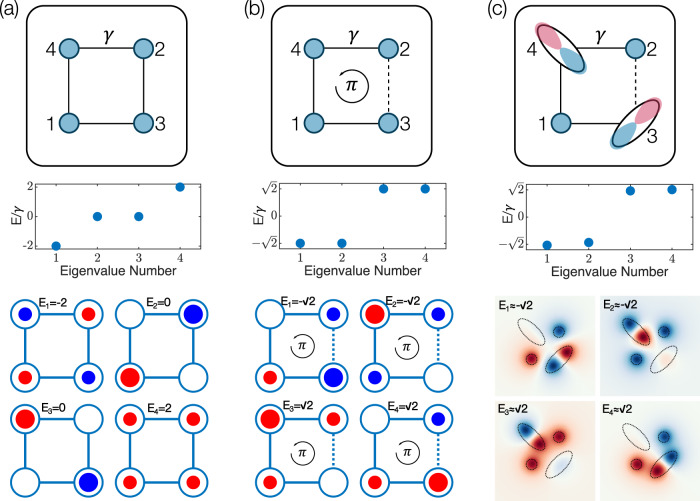
Synthetic *π* flux threading a unit cell plaquette induced by *s* and *p* orbitals. Schematic and corresponding eigenvalues and eigenmodes of a unit cell plaquette **a** composed of only *s* orbitals without synthetic *π* flux, **b** with synthetic *π* flux, and **c** composed of both *s* and *p* orbitals, respectively. The solid and dashed lines in the schematic indicate the positive and negative couplings, respectively. *E* denotes the eigenvalues and *γ* denotes the coupling rate between the nearest-neighbor sites. The areas and colors of the circles indicate amplitudes and phases of the corresponding eigenmodes, respectively. As can be seen in **a** and **b**, the artificial introduction of a *π* flux leads to a significant change in the eigenspectrum and eigenmodes. When a wavefunction in **c** crosses the *p* orbital at site 3 a *π*-phase is accumulated, thereby inducing a flux in the plaquette. This effect is confirmed by the fact that the eigenvalues and eigenmodes from the full continuum calculations (**c**) line up with the states in **b**.

Here, we present a QTI in a photonic system that uses the symmetry representations of on-site orbitals to generate the necessary synthetic *π* fluxes. We consider *s* and *p* orbitals, which have inherent even and odd parities, respectively. We exploit the property that, as the wavefunctions of the collective system traverse a *p* orbital, they accumulate a phase of *π*^9^. The combination and judicious control of the orbitals in a four-site unit cell [Fig. 1c], breaking the symmetries of mixed-orbital lattices, creates a synthetic *π* flux that opens a gap at "half-filling" which, along with the modulation in the hopping amplitudes, results in the gapped system having a QTI phase. We experimentally demonstrate the photonic QTIs in a waveguide lattice fabricated using direct laser writing by showing the existence of mid-gap modes, which are localized at the corners of the lattice. The fabrication of the waveguides by direct laser writing allows for unprecedented control over the waveguide parameters including both the cross-sections and the trajectories of the waveguides^10,29^, which was not straightforward in the conventional femtosecond direct laser writing technique^30^.

## Results

We start by presenting the implementation of our QTI by using *s* and *p* orbitals to induce *π* flux threading a unit cell, taking advantage of the *π*-phase accumulated by the wavefunction as it crosses the *p* orbital. A square unit cell is composed of four sites with different orbitals: two *p* orbitals, which have the major axes tilted from the *y*-axis by ± 45^∘^, respectively, and two *s* orbitals as shown in Fig. 1c. We note that the coupling strength ∣*γ*∣ between any adjacent pair of *s* and *p* orbitals inside the unit cell is identical. However, the lobe of the *p* orbital that couples more strongly with the *s* orbital is dictated by its corresponding spatial proximity to the lobe (i.e., formally known as the overlap integral), resulting in a non-zero net coupling between the sites. It can then be seen, as in Fig. 1c, that for any wavefunction that loops around a unit cell plaquette there must be exactly one traversal of a *p* orbital through both lobes so that a "sign flipping” occurs, which effectively produces a negative coupling. Since the flux threading a unit cell is defined as the accumulated phase of the wavefunction that loops around a unit cell plaquette, the resulting flux induced in the system has a value of *π*. We first validate that the *π* flux threads through this square unit cell by comparing three unit cell plaquettes as shown in Fig. 1: those composed of only *s* orbitals without and with *π* flux, and a unit cell plaquette composed of both *s* and *p* orbitals.

To compare the three unit cell plaquettes, we computed the eigenvalues and eigenmodes of the first two cases, having only *s* orbitals, using the tight-binding Hamiltonians, and the other case, having both *s* and *p* orbitals, using the full-continuum wave equation. For a unit cell plaquette without a *π* flux [Fig. 1a], the eigenvalues are −2*γ*,0,2*γ*, where only the zero-energy states are twofold degenerate. Whereas for a unit cell plaquette, in that a *π* flux threading the plaquette is induced by introducing a negative coupling [Fig. 1b], the eigenvalues are $\pm \sqrt{2}\gamma$, each of which is twofold degenerate. On the other hand, the eigenvalues and eigenmodes of the unit cell plaquette with both *s* and *p* orbitals calculated using the full-continuum wave equation show a great resemblance with the unit cell with the synthetic *π* flux [Fig. 1c], where the bulk gap is opened. The similarity between these two unit cell plaquettes validates that the specific arrangement of having two *p* orbitals in the same unit cell aligned at different angles induces the effective magnetic flux of *π* per plaquette.

**Fig. 2:**
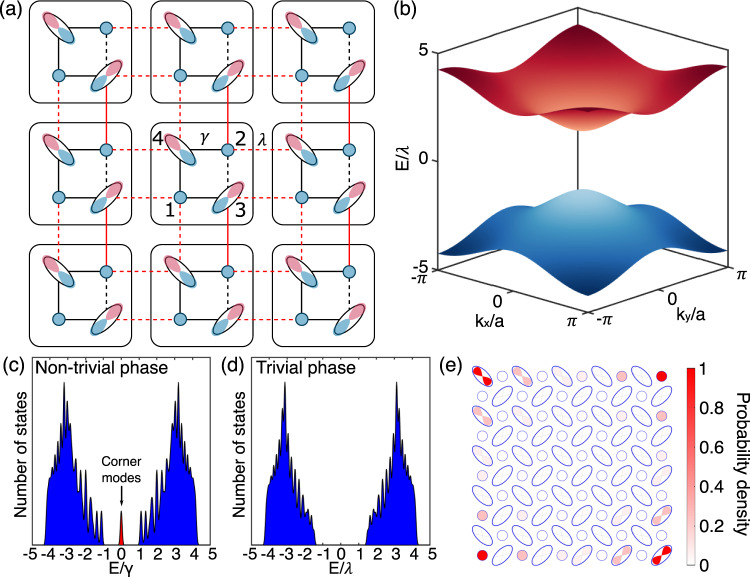
Quadrupole topological insulator using orbital-induced synthetic flux. **a** Schematic of the quadrupole topological insulator with orbital-induced synthetic flux. For the tight-binding model, *E* denotes the eigenvalues, and *γ* and *λ* are the nearest-neighbor coupling terms within (black) and across (red) unit cells, respectively. Dashed lines represent coupling terms with negative signs due to the overlap with the "negative part'' of the *p* orbital. Numbers indicate the basis of the Hamiltonian. **b** A bulk band structure where ∣*γ*/*λ*∣ = 1/2. The band structure consists of two twofold degenerate bands. **c** The numerically calculated density of states in the nontrivial phase (∣*γ*/*λ*∣ = 1/2) and **d** trivial phase (∣*γ*/*λ*∣ = 2), respectively, where the system has 10 × 10 unit cells. **e** Combined eigenmode local density of states of the four topologically protected corner modes in the nontrivial phase (∣*γ*/*λ*∣ = 1/2). Here, the system has 5 × 5 unit cells as in the experiment.

For the tight-binding description of the model, we choose a base where all *s* orbitals have a phase of zero and one fixed site of the *p* orbital has a phase of zero while the opposite site has a phase of *π*. In Fig. 1c and Fig. 2a, zero and *π* phases of the basis states are colored in blue and red, respectively. As the hopping is determined by the overlap of the base states, the "negative part” of the *p* orbital results in some negative hoppings. Then, the tight-binding bulk Hamiltonian of this system becomes:

1 $${h}^{oq}({{{{{{{\bf{k}}}}}}}},\delta )=	\left[\gamma -\lambda \cos ({k}_{x}a)\right]{{{\Gamma }}}_{4}+\lambda \sin ({k}_{x}a){{{\Gamma }}}_{3}\\ 	+\left[\gamma -\lambda \cos ({k}_{y}a)\right]{{{\Gamma }}}_{2}+\lambda \sin ({k}_{y}a){{{\Gamma }}}_{1}+\delta {{{\Gamma }}}_{0},$$

where *a* is the lattice constant, *γ* and *λ* are the nearest-neighbor coupling terms within and across unit cells, respectively, the Γ-matrices^18^ are Γ_*j*_ = − *τ*_2_⨂*σ*_*k*_ for *j* ∈ {1, 2, 3}, Γ_0_ = *τ*_3_⨂*σ*_0_, Γ_4_ = *τ*_1_⨂*σ*_0_ where *τ* and *σ* are Pauli matrices for the degrees of freedom within a unit cell. When the on-site energy of the *s* and *p* orbitals are the same, *δ* = 0. This Hamiltonian closely resembles those Hamiltonians studied in the previous realizations of the quadrupole insulators^18,20,21^, where the only difference is the sign of $\lambda \cos ({k}_{x,y}a)$ in the first and third terms on the right-hand side. The consequent difference is that at the phase transition point in the original model the bandgap closes at the **M** point, while in our model it occurs at the **Γ** point. In addition, this Hamiltonian has two mirror symmetries *M*_*x*_ and *M*_*y*_, which do not commute with each other, and also has *C*_4_ symmetry. Figure 2b shows the 2D bulk band structure of our model in the topologically nontrivial phase, where the bandgap has opened at the **Γ** point due to the synthetic *π* flux in the system. For the system with open boundary conditions, as shown in Fig. 2a, the nontrivial quadrupole phase leads to gapped edge states and in-gap corner states, as shown in Fig. 2c–e (see Supplementary Section I for a more detailed discussion of the symmetries and the topological quadrupole phases of the Hamiltonian).

We experimentally verify the quadrupole topology of the system by considering a two-dimensional lattice of evanescently-coupled waveguides. A square unit cell is composed of four waveguides: two elliptical waveguides, which have the major axes tilted from the *y*-axis by ± 45^∘^, respectively, and two circular waveguides as shown in Fig. 2a. We control the radii of waveguides such that the lowest-energy mode (*s* orbital) of the circular waveguides and the second-lowest-energy mode (*p* orbital) of the elliptical waveguides have the same propagation constant, enabling them to couple with one another. The lowest-energy mode of the elliptical waveguides has a propagation constant far detuned from the other modes, such that they are well separated from our main system and can be neglected (see Supplementary Section II for the Eigenmode calculations with the full-continuum Hamiltonian).

The equation governing the diffraction of light through the waveguide array is:

2 $$i{\partial }_{z}\psi ({{{{{{{\bf{r}}}}}}}},\, z)=\hat{H}\psi ({{{{{{{\bf{r}}}}}}}},\, z),$$

where *ψ*(**r**, *z*) is the transverse electric field amplitudes at propagation distance *z*. $\hat{H}$ is the wavelength (*λ*) dependent continuum Hamiltonian for the wave propagation in the waveguide array. Since we only consider a single bound mode for each waveguide and it evanescently couples to the neighboring waveguides, we can approximate the diffraction of light in our waveguide array using a tight-binding model, *i*∂_*z*_*ψ*_*i*_(*z*) = − ∑_*j*_*c*_*ij*_(*λ*)*ψ*_*j*_(*z*), where *ψ*_*i*_ is the amplitude in the *i*-th waveguide, and *c*_*ij*_(*λ*) is the coupling constant between waveguides *i* and *j* at wavelength *λ*.

**Fig. 3:**
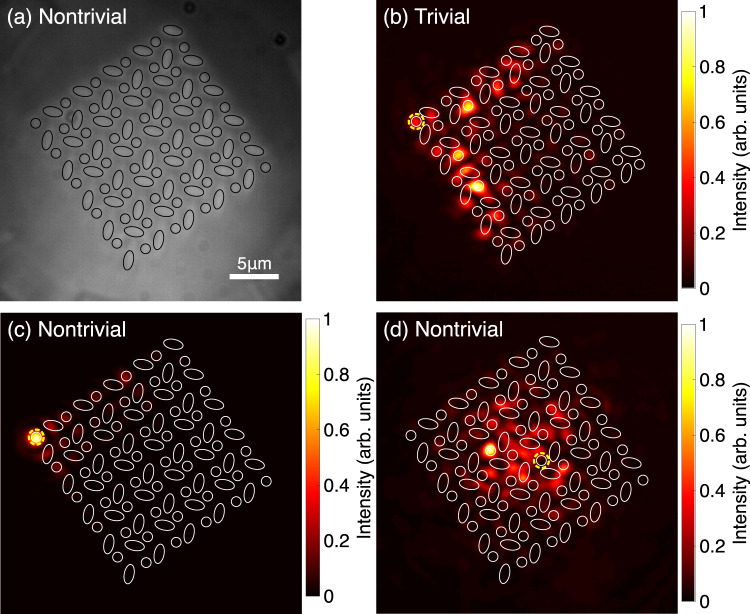
Experimentally measured diffracted light at the output facet. **a** Cross-sectional image of the output facet of the waveguide array in the nontrivial phase with a broad illumination of the input facet (see Supplementary Fig. S3). **b**–**d** Measured intensity profiles at the output facet of the waveguide structures. Waveguides, where light is injected at the input facet, are indicated with yellow dashed circles. The intensity profiles are normalized to their respective maximum value to increase visibility. **b** Light is injected into the waveguide at the left corner of the waveguide array in the trivial phase and **c** nontrivial phase, respectively. **d** When light is injected into a waveguide in the center of the waveguide array in the nontrivial phase, it spreads into the bulk of the structure. In **a** and **b**–**d**, black and white lines are overlapped to indicate the positions of the waveguides, respectively.

To experimentally observe the corner-localized topological modes, the light was injected into a waveguide at one of the corners of the waveguide array. In Fig. 3b, c, the diffracted light observed from the output facet for two different topological phases is shown. When the waveguides located at the corners of the waveguide array are excited in a trivial phase, the injected light spreads significantly into the bulk [Fig. 3b], which indicates that there is no corner-localized eigenmode. On the other hand, for the nontrivial phase, the light does not diffract into the bulk and is tightly confined close to the corner where the light was initially injected [Fig. 3c]. This confinement of the light at the injected corner is an indication of the presence of the corner modes and their localization is due to the nontrivial topology of the system. To prove further that the spatial localization of the corner modes in the nontrivial phase is not due to the weak coupling between the waveguides, light is injected at a waveguide in the center of the waveguide array. The injected light diffracts significantly into the bulk of the structure as shown in Fig. 3d, which supports further that the corner-localized mode shown in Fig. 3c emerges due to the nontrivial topology of the model.

**Fig. 4:**
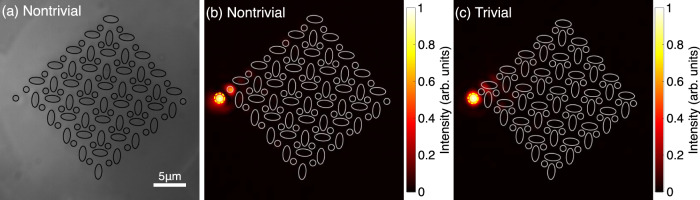
Direct excitation of the corner mode using an auxiliary waveguide weakly coupled to the system. **a** Cross-sectional image of the output facet of the waveguide array in the nontrivial phase with an auxiliary waveguide with a broad illumination of the input facet (see Supplementary Fig. S3). **b** Diffracted light measured at the output facet when light is injected into the auxiliary waveguide directly at the left corner of the waveguide array in the nontrivial phase and **c** trivial phase, respectively. Waveguides, where light is injected at the input facet, are indicated with yellow dashed circles. The intensity profiles are normalized to their respective maximum value to increase visibility. In **a** and **b**, **c**, black and white lines are overlapped to indicate the positions of the waveguides, respectively. An analogous experimental measurement of the corner mode localized at the *p*-orbital corner is presented in Supplementary Material section IV. "Observation of p-orbital corner mode".

However, the localization of the corner modes alone does not prove the quadrupole properties, as this behavior was also observed in a system similar to ours, just without a *π* flux. In this system, however, the corner state lies not in a bandgap but is a bound state in the continuum^31^. To demonstrate that the corner localized modes in our system are indeed in bandgap and topologically nontrivial modes due to the quadrupole topology, we introduce auxiliary waveguides as shown in Fig. 4a. The auxiliary waveguides are weakly coupled to the lattice such that they can be used as an external drive injecting light into the lattice at the energy of their bound modes without significantly altering the intrinsic modes of the lattice^19^. In the experiment, the center-to-center distance from the auxiliary waveguide to the waveguide at the corner is 2.1 μm. The auxiliary waveguide is identical to the circular waveguides in the lattice, therefore the energy of the light injected from the auxiliary waveguide into the lattice is at zero energy. In our system, the light initially injected at the auxiliary waveguide couples only to the corner state in the nontrivial phase [Fig. 4b] but does not couple into the system in the trivial phase [Fig. 4c]. An analogous experimental measurement of the corner mode localized at the *p*-orbital corner is presented in Fig. S4 in Supplementary Material section IV. "Observation of p-orbital corner mode". This proves experimentally that in our system with *s* and *p* orbitals, the *π* flux is induced in the unit cell, which opens the bandgap, and that the corner localized modes in the nontrivial phase are pinned at midgap due to the quadrupole topology of the system.

## Discussion

In this work, we have demonstrated synthetic crystalline structures composed of multiple orbitals. We realized the quadrupole topological insulator in which synthetic *π* flux threading each plaquette is induced due to the different symmetry representations of the orbitals. To prove the nontrivial quadrupole topology of the system, we have experimentally verified that our realization of the quadrupole topological insulator has zero-dimensional corner-localized modes in the middle of the band gap. As a very unique feature, this approach results in two distinct types of topologically protected corner modes having different symmetries of *s* and *p* orbitals that naturally extend to interesting applications that exploit those symmetries. Previously, the orbital degree of freedom has been nearly exclusively utilized in the ultracold atoms in optical lattices among diverse metamaterial systems. Different experimental platforms have their unique features, such as abilities to more precisely or actively control the gain/loss, on-site energies, or coupling strengths, which can be associated with the orbital degree of freedom to open the possibility to study richer physics. Our method of incorporating different orbitals could be extended into 3D to generate a quantized octupole insulator, by leveraging *d* and *s* orbitals in a cubic unit cell. Alternatively, by appropriate modulation of the on-site energy and coupling as a function of propagation distance (*z*), a dipole pumping could be realized, which can be interpreted as a topological insulator with hinge-localized chiral modes in (2+1)D^28^. Furthermore, the realization of such photonic quadrupole topological insulators in the time-reversal symmetric system can provide a more straightforward route to utilize the quadrupole topology in practical applications, since the geometry we present is easier to implement than the one proposed previously.

## Methods

The radii of the major and minor axes of the elliptical waveguides in the experiment are 0.6 μm and 1.3 μm, and the radius of the circular waveguides is 0.5 μm. The center-to-center distances between the waveguides determine the coupling strength, which in our structure are dimerized to be 1.6 μm and 2.1 μm for strong and weak couplings, respectively. In our experimental system, the coupling strength decreases exponentially with increasing separation between waveguides. Although the longer-range couplings cannot be removed completely, the strength of the next-nearest-neighbour coupling is sufficiently weak that the aforementioned tight-binding description of the system is a good approximation for our experimental system (for more detail, see Supplementary Section II for the Eigenmode calculations with the full-continuum Hamiltonian). The core of the waveguide is made out of the resin SU8-2 (Microchem) with a refractive index of ${n}_{{{{{{{{\rm{core}}}}}}}}}=1.59$ and is surrounded by IP-Dip (Nanoscribe), which has a refractive index of *n*_clad_ = 1.54. The sample was fabricated using a Nanoscribe Photonic Professional GT^29,32–34^ (see Supplementary Section III for Details about the Fabrication). For the measurements, light with a wavelength of 760 nm from a white light laser (NKT photonics with VARIA filter box) is injected with a 20× objective (NA = 0.4) to a selected waveguide at the input facet of the waveguide array. After the propagation through the 1 mm long structure, the diffracted light at the output facet is imaged with another 20× objective onto a CMOS camera (Thorlabs DDC1545M) [the measurement setup is sketched out in Fig. S3a].

## Supplementary information

Supplementary Information

## Data Availability

The data that support the findings of this study are available from the corresponding author upon reasonable request.
