# Supplementary material for: Photonic quadrupole topological insulator using orbital-induced synthetic flux
Source: Nat Commun. 2022 Nov 3;13:6597. doi: 10.1038/s41467-022-33894-6 (PMC9633597; doi:10.1038/s41467-022-33894-6)
Supplement: Supplementary file 1 — Supplementary Information [file 41467_2022_33894_MOESM1_ESM.pdf]

Supplemental Materials: Photonic quadrupole topological insulator using orbital-induced synthetic flux

Julian Schulz†,[1] Jiho Noh†,[2] Wladimir A. Benalcazar,[3,4]
Gaurav Bahl,[2] and Georg von Freymann[1,5]

[1]*Physics Department and Research Center OPTIMAS, TU Kaiserslautern, 67663 Kaiserslautern, Germany*

[2]*Department of Mechanical Science and Engineering, University of Illinois at Urbana–Champaign, Urbana, IL 61801 USA*

[3]*Department of Physics, Princeton University, Princeton, New Jersey 08542, USA*

[4]*Department of Physics, Emory University, Atlanta, Georgia 30322, USA*

[5]*Fraunhofer Institute for Industrial Mathematics ITWM, 67663 Kaiserslautern, Germany*

†These authors contributed equally

e-mail: schulzj@rhrk.uni-kl.de; jihonoh@illinois.edu; wladimir.benalcazar@gmail.com; bahl@illinois.edu; georg.freymann@physik.uni-kl.de

I. Quadrupole phases

We discuss the bulk properties of the model by imposing the periodic boundary condition on a unit cell. The symmetry group, that protects the quantization of both components of the polarization p_x and p_y and the quadrupole moment q_{xy}, includes two mirror symmetries $M_x = \tau_3 \bigotimes \sigma_1$ and $M_y = \tau_1 \bigotimes \sigma_1$ that do not commute with each other [S1]. When $\delta = 0$, the Hamiltonian (Eq. 1) has the required mirror symmetries, $M_x h^{oq}(k_x, k_y) M_x^\dagger = h^{oq}(-k_x, k_y)$ and $M_y h^{oq}(k_x, k_y) M_y^\dagger = h^{oq}(k_x, -k_y)$.

These two mirror symmetries satisfy the condition for the quantization of the quadrupole moment such that they do not commute with each other and satisfy $\{M_x, M_y\} = 0$. In addition, the Hamiltonian (Eq. 1) has C_4 and chiral symmetries, $C_4 h^{oq}(k_x, k_y) C_4^\dagger = h^{oq}(k_y, -k_x)$ and $\mathcal{C} h^{oq}(k_x, k_y) \mathcal{C}^\dagger = -h^{oq}(k_x, k_y)$, where $C_4 = \{(\tau_1 + i\tau_2) \bigotimes \sigma_0 - (\tau_1 - i\tau_2) \bigotimes i\sigma_2\}/2$ and $\mathcal{C} = \tau_3 \bigotimes \sigma_0$. The bulk Hamiltonian is gapped for $|\gamma/\lambda| \neq 1$ but closes at $\mathbf{\Gamma}$ point when $|\gamma/\lambda| = 1$, where the topological transition occurs. Since the inversion symmetry $\mathcal{I}$ is related to the mirror symmetries as $\mathcal{I} = M_y M_x$, the Hamiltonian is also inversion symmetrical.

We further study the quadrupole topology from the Bloch Hamiltonian by considering the Wannier bands and their polarization through the nested Wilson loop formulation [S1]. The Wannier centers $\nu_x^j(k_y)$ are proportional to the phases of the eigenvalues of the Wilson loop operator, $\mathcal{W}_x$ [Fig. S1(a)], and the polarization p_x can be obtained by taking the integral of the Wannier bands over the whole Brillouin zone in the y-direction. The polarization p_y can be determined similarly by considering $\mathcal{W}_y$ and the corresponding $\nu_y^j(k_x)$ [Fig. S1(b)]. Note that for the Bloch Hamiltonian in our model, the Wannier bands are gapped and $p_x = p_y = 0$, which indicates that the bulk dipole moments are zero. Here, we denote the upper and lower Wannier bands as $\nu_x^\pm$ ($\nu_y^\pm$), respectively. Using the nested Wilson loop method, we can also compute the polarizations of each Wannier band. The aforementioned existence of non-commuting mirror symmetries $M_{x,y}$ quantizes the Wannier-sector polarizations $p_x^{\nu_\pm}$ and $p_y^{\nu_\pm}$ to be either 0 or 1/2, which subsequently quantizes the bulk quadrupole $q_{xy} = 2p_x^{\nu_\pm} p_y^{\nu_\pm}$ to be either 0 or 1/2. From the calculation we find that $p_{x,y} = 0$ for $|\gamma/\lambda| \neq 1$. On the other hand, we find that $q_{xy} = 0$ for $|\gamma/\lambda| > 1$ but $q_{xy} = 1/2$ for $|\gamma/\lambda| < 1$, which proves the quadrupole topology of the model.

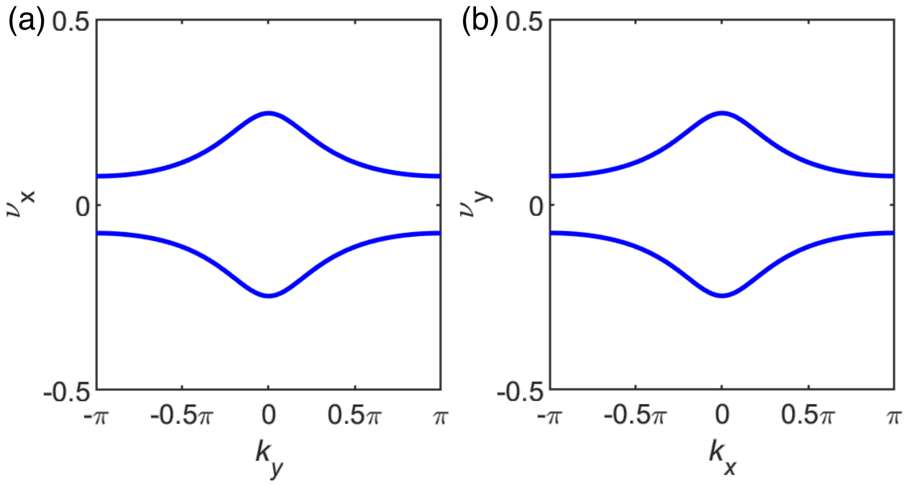

FIG. S1. (a) Wannier bands $\nu_x(k_y)$ and (b) $\nu_y(k_x)$ computed for the bottom two bands of the bulk band structure where $\gamma/\lambda = 1/2$.

II. Eigenmode calculations using the full-continuum Hamiltonian

While studying the evanescently-coupled waveguide system for realizing the photonic quadrupole topological insulators, we only considered the case where the lowest-energy mode (s orbital) of the circular waveguides and the second-lowest-energy mode (p orbital) of the elliptical waveguides have the same energy. The lowest-energy mode of the elliptical waveguide is ignored in the model since due to the judiciously controlled waveguide radii, the energy of this mode is well separated from the other modes of interest. To confirm this, we calculated the eigenmodes using the full-continuum calculation by diagonalizing a continuum Hamiltonian for the propagation of the wave in the photonic lattice (Eq. 2). In Fig. S2, we show the eigenmodes of the 5×5 unit cell quadrupole topological insulator with orbital-induced synthetic flux, identical to the experiment. As shown in Fig. S2, the energy of the bulk band consisting of the lowest-energy-mode of the elliptical waveguide is spectrally well separated from the eigenmodes consisting of both s orbital and p orbital.

In the bands of interest, the eigenenergies are not symmetric with respect to the mid-gap as expected in a chiral symmetric system since the chiral symmetry of the system is broken due to the non-zero long-range hoppings. Although the hopping strength decays exponentially as a function of spatial separation between the waveguides, the long-range hoppings between the waveguides cannot be removed completely. However, in our work, the amount of the deviation from the perfectly chiral symmetric system is small enough

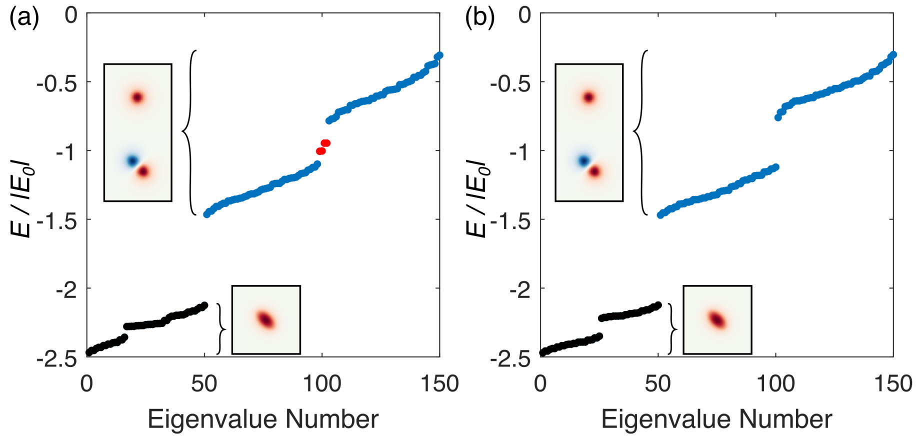

FIG. S2. (a) Eigenenergies of the 5×5 unit cell waveguide array in the non-trivial phase and (b) trivial phase, respectively. Eigenmodes are calculated using the full-continuum calculation by diagonalizing a continuum Hamiltonian for the propagation of the wave in the photonic lattice. Black dots indicate the bulk eigenmodes composed of the lowest-energy-mode of the elliptical waveguide. Blue(red) dots indicate the bulk(corner) eigenmodes composed of the lowest-energy mode of the circular waveguide (s orbital) and the second-lowest-energy mode (p orbital) of the elliptical waveguides.

that we assume that the system closely follows the tight-binding model. As a measure of the degree of chiral symmetry breaking in the topological phase due to the next-nearest-neighbor coupling, we consider the ratio of the nearest-neighbor coupling across the unit-cell ($c_{\text{n.n.}}$) and the next-nearest-neighbor coupling within the unit-cell ($c_{\text{n.n.n.}}$). Based on the continuum simulation, this ratio is found to be $c_{n.n.n.}/c_{n.n.} \approx 0.05$.

III. Details on the sample fabrication

The sample was fabricated using the Nanoscribe Photonic Professional GT similar to the way laid out in [S2]. First, the structure is 3D-printed by two-photon lithography in a negative-tone photoresist (IP-Dip, Nanoscribe). After the development (1 h in Propylene glycol methyl ether acetate and 1 h in Isopropanol) one is left with the inverse waveguide structure like the one shown in Fig. S3(d). A surrounding support grid is rotationally symmetrically designed and written with higher laser power to minimize distortion of the waveguide structure due to shrinkage of the photoresist during development. The next step is to dip the structure in gamma-butyrolactone. After one hour, where the structure is left to soak in the solvent, most of the gamma-butyrolactone is removed so that only the structure is wetted. A drop of SU8-2 (MicroChem) is then placed on the structure so that

the SU8 can diffuse into the channels filled with gamma-butyrolactone. Finally, the sample is slowly heated up ($10\,\mathrm{K\,min^{-1}}$) with a hotplate at $150\,^{\circ}\mathrm{C}$ for $5\,\mathrm{min}$ to solidify the SU8. The pre-infiltration with gamma-butyrolactone helps to increase the likelihood that the thin channels will be fully infiltrated, as the SU8 enters the channels by diffusion. Otherwise, the infiltration process would only rely on capillary forces, which can be hindered, e.g., by thin polymer threads. However, the SU8 should be diluted as little as possible with gamma-butyrolactone because it decreases in volume when baked out, allowing it to retract into the channels. After the baking process, the channels are filled with a material with a higher refractive index as IP-Dip and become waveguides and look brighter than the surrounding material [see Fig. S3(b,c)].

IV. Observation of p orbital corner mode

In the main text, we have presented the proof of induced synthetic π flux threading the system by exploiting the different symmetry representations of the orbitals. To prove this we have devised two different experiments: (1) direct injection of the light at the corner waveguide proved that there exist corner localized modes due to the non-trivial topology of the system and (2) an injection of the light through an auxiliary waveguide proved that there exists a bandgap due to the π flux threading the system and that the corner localized modes are at midgap. In both experiments, the light was injected at the corner, where the corner localized mode was an s orbital in a circular waveguide. Although not presented in the main text, the same experiments can be done at the corners, where the corner localized mode is a p orbital in an elliptical waveguide as predicted in the model. We performed the experiment in which we inject light into the system through an auxiliary waveguide. Just as in the case when the corner mode was an s orbital, the light initially injected at the auxiliary waveguide does not couple into the system in the trivial phase, but couples only to the corner state in the non-trivial phase, where we can clearly see the shape of the p orbital having two lobes with a node at the center.

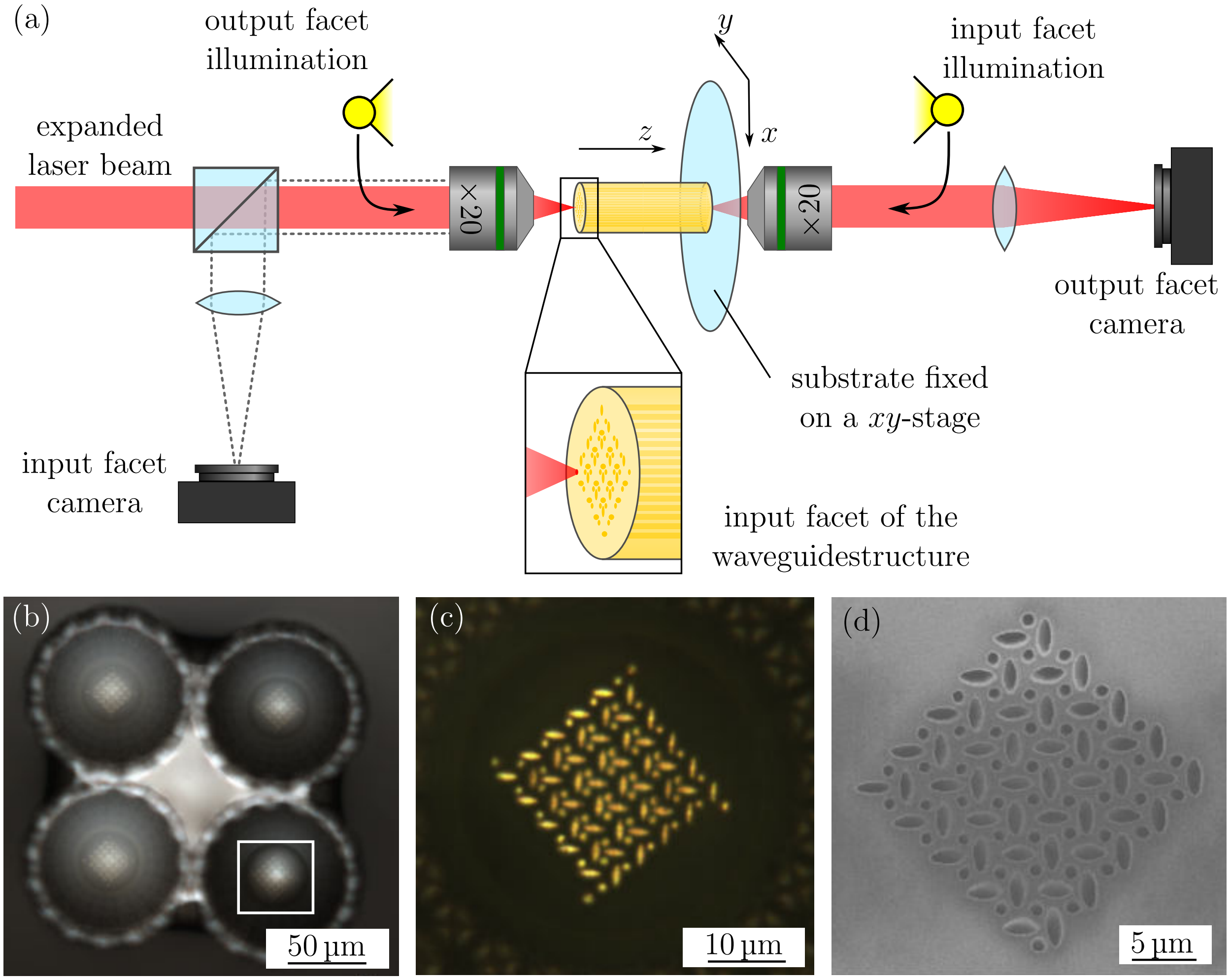

FIG. S3. (a) Schematic of the measurement setup. To image the input or output facet on the cameras a light-emitting diode is put into the beam path to serve as transmission illumination. (b) Microscope image of the input facet in reflection illumination of a final structure. Only after the baking process, the channels become waveguides and look brighter than the surrounding material. (c) Microscope image of the input facet in transmission illumination of the waveguide array marked in (b) with a white box. (d) SEM Image of an inverse waveguide structure after development before the infiltration.

References

[S1] W. A. Benalcazar, B. A. Bernevig, and T. L. Hughes, Quantized electric multipole insulators, Science **357**, 61–66 (2017).

[S2] J. Schulz, S. Vaidya, and C. Jörg, Topological photonics in 3D micro-printed systems, APL Photonics **6**, 080901 (2021).

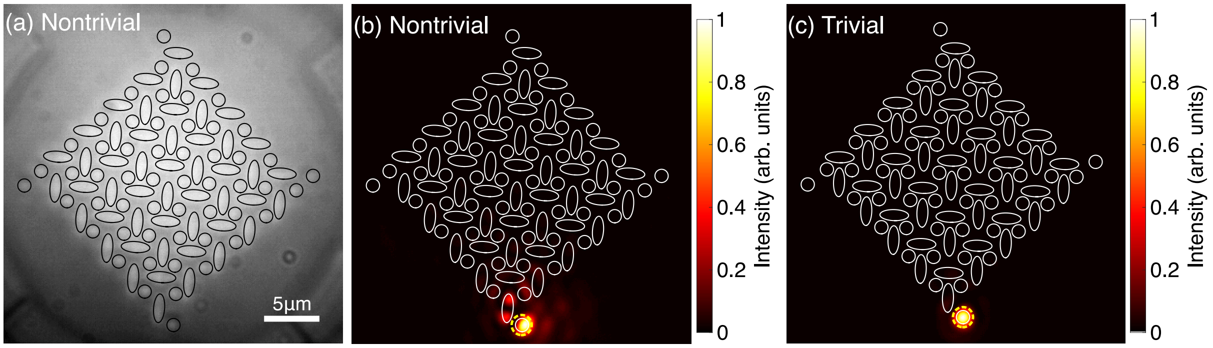

FIG. S4. **Direct excitation of the corner p-mode using an auxiliary waveguide weakly coupled to the system.** (a) Cross-sectional image of the output facet of the waveguide array in the non-trivial phase with auxiliary waveguides with a broad illumination of the input facet. (b) Diffracted light measured at the output facet when light is injected into the auxiliary waveguide directly at the lower corner of the waveguide array in the non-trivial phase after 300 µm propagation and (c) trivial phase after 1000 µm propagation, respectively. Waveguides, where light is injected at the input facet, are indicated with yellow dashed circles. The intensity profiles are normalized to their respective maximum value to increase visibility. In (a) and (b-c), black and white lines are overlapped to indicate the positions of the waveguides, respectively.
